# Artificial intelligence-based internet hospital pharmacy services in China: Perspective based on a case study

**DOI:** 10.3389/fphar.2022.1027808

**Published:** 2022-11-09

**Authors:** Fengjiao Bu, Hong Sun, Ling Li, Fengmin Tang, Xiuwen Zhang, Jingchao Yan, Zhengqiang Ye, Taomin Huang

**Affiliations:** ^1^ Department of Pharmacy, Eye & ENT Hospital, Fudan University, Shanghai, China; ^2^ Information Center, Eye & ENT Hospital, Fudan University, Shanghai, China

**Keywords:** internet hospital, artificial intelligence, prescription preview, medication pick-up code, online medication consultation

## Abstract

**Background:** Recently, internet hospitals have been emerging in China, saving patients time and money during the COVID-19 pandemic. In addition, pharmacy services that link doctors and patients are becoming essential in improving patient satisfaction. However, the existing internet hospital pharmacy service mode relies primarily on manual operations, making it cumbersome, inefficient, and high-risk.

**Objective:** To establish an internet hospital pharmacy service mode based on artificial intelligence (AI) and provide new insights into pharmacy services in internet hospitals during the COVID-19 pandemic.

**Methods:** An AI-based internet hospital pharmacy service mode was established. Initially, prescription rules were formulated and embedded into the internet hospital system to review the prescriptions using AI. Then, the “medicine pick-up code,” which is a Quick Response (QR) code that represents a specific offline self-pick-up order, was created. Patients or volunteers could pick up medications at an offline hospital or drugstore by scanning the QR code through the window and wait for the dispensing machine or pharmacist to dispense the drugs. Moreover, the medication consultation function was also operational.

**Results:** The established internet pharmacy service mode had four major functional segments: online drug catalog search, prescription preview by AI, drug dispensing and distribution, and AI-based medication consultation response. The qualified rate of AI preview was 83.65%. Among the 16.35% inappropriate prescriptions, 49% were accepted and modified by physicians proactively and 51.00% were passed after pharmacists intervened. The “offline self-pick-up” mode was preferred by 86% of the patients for collecting their medication in the internet hospital, which made the QR code to be fully applied. A total of 426 medication consultants were served, and 48.83% of them consulted outside working hours. The most frequently asked questions during consultations were about the internet hospital dispensing process, followed by disease diagnosis, and patient education. Therefore, an AI-based medication consultation was proposed to respond immediately when pharmacists were unavailable.

**Conclusion:** The established AI-based internet hospital pharmacy service mode could provide references for pharmacy departments during the COVID-19 pandemic. The significance of this study lies in ensuring safe/rational use of medicines and raising pharmacists’ working efficiency.

## Introduction

Telemedicine services are a growing technology that promotes quality in healthcare worldwide (Qiu et al., 2018; Bokolo, 2021). With the increasing use of internet and decreasing cost of telemedicine services, the internet hospital has greatly impacted many developing countries where healthcare services are concentrated in large cities (Tu et al., 2015). Ever since “Opinion on promoting the development of internet + medical and health care” was issued by the state council of the People’s Republic of China (PRC) (The State Council of the People’s Republic of China, 2018), internet plus healthcare centers have been emerging. These centers significantly saved patients’ time and economic costs and played a crucial role in the prevention and control of COVID-19 epidemic (Li et al., 2020; Gong et al., 2020; Sun et al., 2020). As patients’ medical behavior patterns are shifting from on-site to online, the demand for internet hospitals has increased (Ge et al., 2022). According to incomplete statistics, over 1,600 hospitals in China obtained the business license of internet hospital by June 2021 (Zhang, 2022). There are three types of internet hospitals in China—those independently operated by entity medical institutions (e.g., public hospitals and private hospitals), government-sponsored internet hospitals, and those sponsored by internet medical enterprises (e.g., Hao Daifu, WeMed, and Ping An Good Doctor, *etc.*) (Han et al., 2020; Jiang et al., 2021; Xu et al., 2021). A cross-sectional survey showed that Chinese patients were more likely to use public hospital-sponsored internet hospitals (Liu and Shi, 2021). Therefore, China’s internet hospitals gradually formed a model that entity hospitals led along with technology companies providing the main technical support under the general trend of government management and patients’ choice. Moreover, pharmacy services acted as a link between doctors and patients, which is essential in improving patient satisfaction, assuring safe and rational drug use, *etc.*

Eye & ENT Hospital of Fudan University is a grade 3A specialized hospital in Shanghai that represents the advanced medical level in China. Its otolaryngology has been ranked number one in the specialty rankings for 11 consecutive years and ophthalmology has been firmly in the top three. The pharmacy service of Eye & ENT Hospital of Fudan University has been at the forefront of the internet + services. However, the existing pharmacy service mode of internet hospitals is primarily manual, making it cumbersome, inefficient, and high-risk. In March 2022, Shanghai faced the most severe test after the normalization of prevention and control of the COVID-19 epidemic. Therefore, there is an urgent need to build an AI-based internet hospital pharmacy service mode during the COVID-19 pandemic.

To strictly implement the prevention and control of the COVID-19 epidemic and further meet patients’ needs, we leveraged the internet hospital services, including online follow-up, dispensing, “offline pick-up” and “logistics delivery,” and online patient medication consultation. Additionally, to ensure patients’ safe, fast, convenient, and efficient access to pharmacy services, we combined pharmacy services in conjunction with artificial intelligence (AI), which simultaneously provided new insights for internet hospitals during the COVID-19 pandemic.

## Meterials and methods

### Design of the AI-based internet hospital pharmacy services

This study was carried out at Eye & ENT Hospital of Fudan University. In April 2020, its internet hospital was approved for a business license and officially launched on 23 June 2020. Initially, it was limited to self-pay patients and gradually expanded its range of users by realizing Shanghai medical insurance settlement. Thus far, there have been over 200,000 internet prescriptions, providing pharmacy services to over 100,000 people nationwide in China.

Before launching internet hospital, we learned from the experiences of the top ten hospitals in the 2020 Fudan edition of China’s best comprehensive hospital ranking (Hospital Management and Fudan Institute., 2020; Li et al., 2022). Information was obtained through the official websites, Alipay Life, WeChat and published literature of these hospitals (Li et al., 2020; Li et al., 2020; Hu et al., 2020; Li et al., 2021; Zhi et al., 2021; Zhang, 2022). Thus, the framework of the AI-based internet hospital pharmacy services was concentrated on improving prescription review, convenient drug collection and medication consultation. The data interface was accomplished by our hospital’s information technology department and the corresponding software company.

### Prescription preview with AI

**FIGURE 1:**
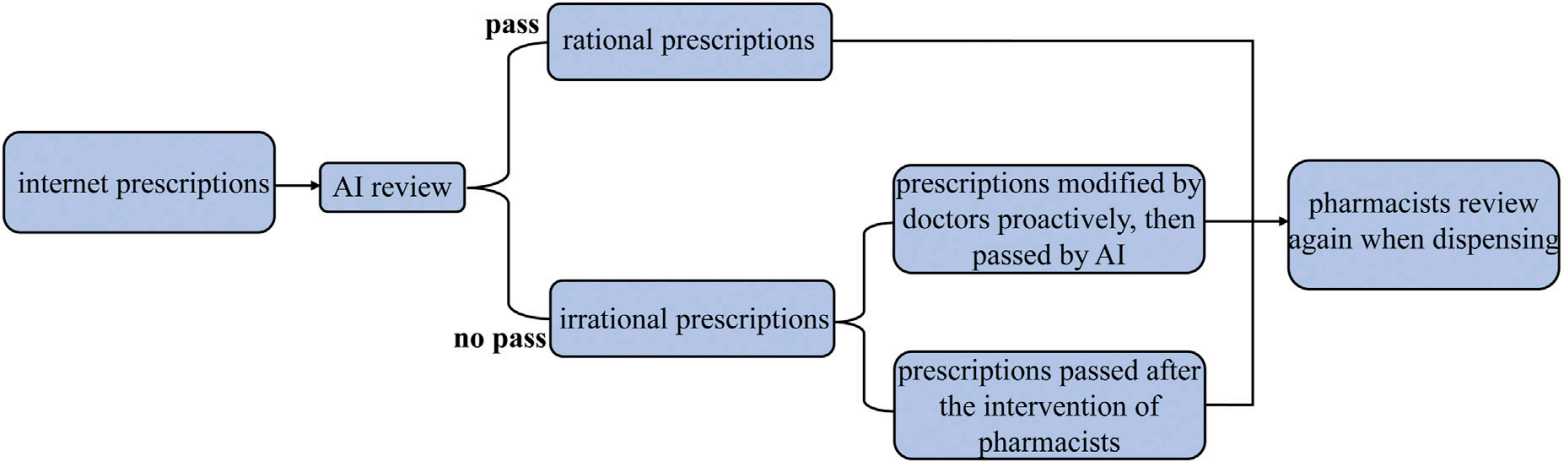
Prescriptions preview process.

The preparation of prescription preview can be traced back to September 2020. First, prescription preview rules were formulated based on drug labels, clinical protocols and guidelines, clinical pathways, national formulary, national prescription laws and regulations *etc.* Then, AI preview was realized using a rational medication monitoring system developed by Beijing Puhua health technology Co., Ltd. The prescription preview process is shown in Figure 1. Three steps were designed to ensure the accuracy of prescriptions, including AI preview, pharmacists preview and double check when dispensing. Moreover, unreasonable prescriptions would be rejected and recorded. Two different levels of problem prescriptions were highlighted, namely, alerts, and interceptions. Absence of a reasonable indication and overdose were defined to be warned, as well as drug-drug interactions and repeated medication. However, those prescriptions cannot be sent when there were any contraindications. After that, the rational medication monitoring system was embedded into the internet hospital system to test the effectiveness of AI-preview. Prescriptions approved by AI were required to be 100% qualified before going live with it.

### Picking up medicine and medicine pick-up code

To ensure the safety and effectiveness of patient medications, refrigerated drugs, fragile drugs, drugs with high risks, and drugs requiring special management and storage requirement at 2–8°C, must be picked up offline based on the “Guidelines for the Construction of Internet Hospitals in Shanghai Municipal Hospitals.” Therefore, the list of drugs that must be picked up offline was made. Other deliverable drugs were entrusted to a third-party pharmaceutical company for delivery. Based on the drug availability in the third-party pharmaceutical company, the drug distribution has two modes: “Large warehouse” and “storage.” The “large warehouse” mode refers to the direct delivery from the warehouse of the pharmaceutical company when the drug is available in the warehouse; the “storage” mode refers to the delivery mode when the drug is unavailable in the warehouse of the pharmaceutical company, and they must pick up drugs from the hospital first and store these drugs in their drugstore. When drugs are dispatched, the logistics information is updated in real-time and patients can check the logistics information on the internet hospital system. Also, the way patients chose to pick up their medications was recorded in the internet hospital. Then the data could be exported in xlsx format and analyzed. Additionally, the idea of quick response (QR) code that represented a specific offline self-pick-up order was proposed on the occasion of zone lockdown in April 2022 and carried out by the information technology department of EENT hospital. The prescription details that directly associated with the offline self-pick-up order number were then written into the QR code and could be read through the dispensing system.

### Medication consultation service

The medication consultation service was supported by Shanghai Liankong network technology Co., Ltd. A volunteer team of licensed pharmacists with extensive clinical experience provided free medication consultation services online. Prior to beginning this service, all pharmacists received standardized training to handle patient questions. If a patient asked questions regarding a disease diagnosis, the pharmacist would guide the patient to consult a clinician. For complex questions, pharmacists would discuss with other pharmacists to ensure that the answers were correct. The basic information of medication consultants and the questions they asked can be recorded, which can be exported to xlsx format. Further analyses were performed to understand the effectiveness of medication counseling.

## Results

### The established AI+ internet hospital pharmacy services mode

**FIGURE 2:**
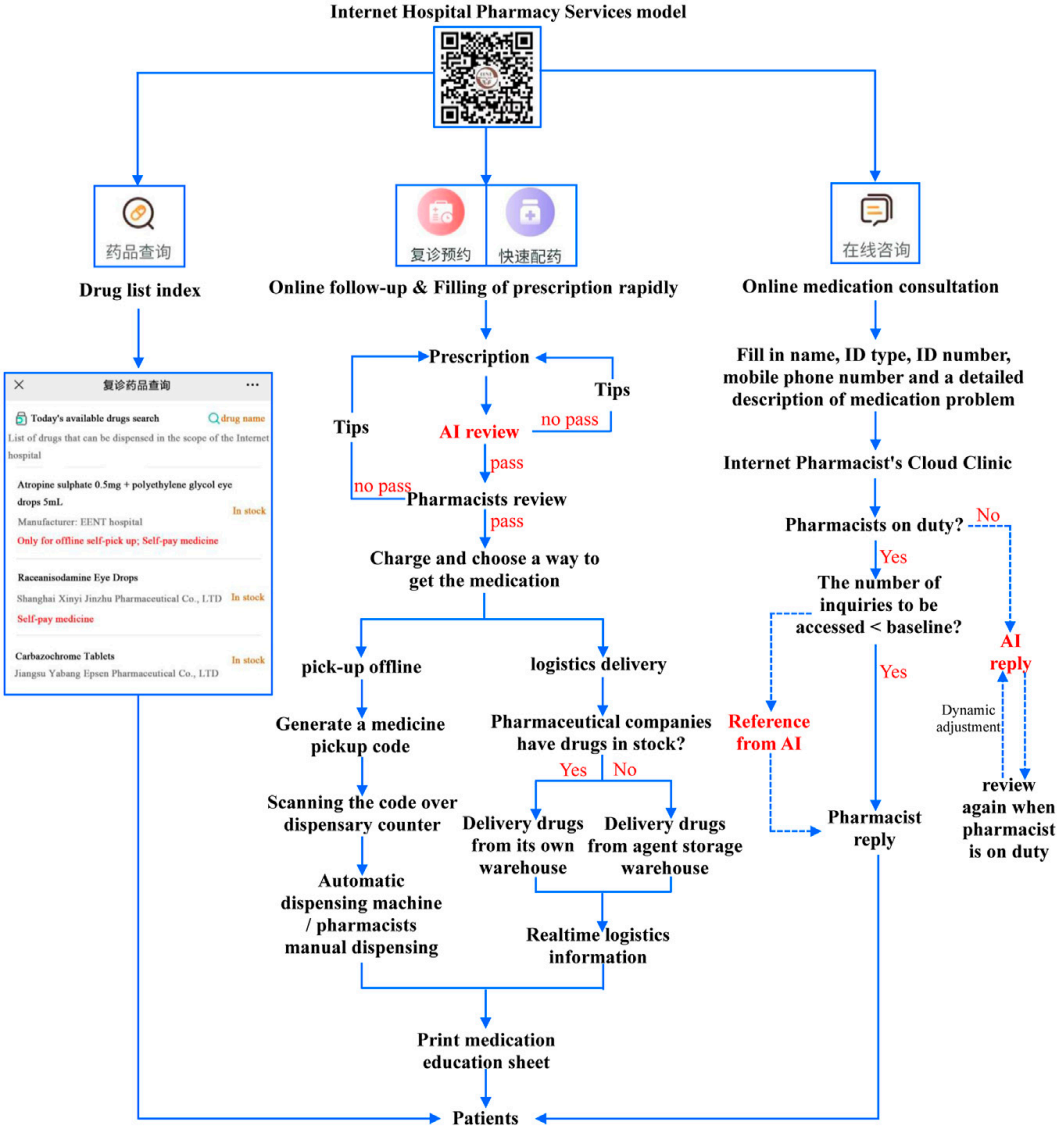
Established AI+ internet hospital pharmacy services mode.

The development of pharmacy service in domestic Internet hospitals was shown in Supplementary Table S1. Only the General Hospital of the Chinese People’s Liberation Army (PLA) was remaining to open its internet hospital services. In the remaining nine hospitals, the preview of prescriptions was carried out manually. All nine hospitals offered patients a variety of choices when it comes to picking up their medication. For online medication consultation, three hospitals provided free services, and six hospitals charged fees according to the pharmacist’s title or the type of consultation. Accordingly, as demonstrated in Figure 2, there were four main pharmacy services in our proposed AI-based internet hospital, including drug list indexing, online follow-up, rapid filling of prescriptions, and online medication consultation. People could search the drug lists online without registering and consulting doctors for a specific drug. Additionally, the generic drug names, manufacturers, and information on the offline pickup drugs were listed in detail.

Patients with medication records in our hospital or other medical institutions in Shanghai in the past 6 months could follow up online, and doctors could then prescribe for them. To ensure safe and rational drug use, the formulated prescription rules were embedded into the internet hospital system to review the prescriptions by AI. There were two steps in the prescription preview process, namely AI automatic and pharmacist manual reviews. Meaning, the prescription was prechecked by AI first, and then AI would tip doctors on whether the prescription was rational. If physicians did not modify their prescriptions accordingly, pharmacists would review the prescription again until the prescriptions were qualified; only the prescriptions that are passed could be charged. After payment, patients could choose a way to pick up their drugs: “Offline self-pick up” or “logistics delivery.” By interconnecting internet hospital patients’ data with social pharmacies, we realized the flow of prescriptions, allowing patients to pick up their medications at a social pharmacy, in addition to picking them up at a hospital pharmacy. Finally, the offline pick-up orders would generate a QR code; the patients or volunteers could pick up medications at the offline hospital or social pharmacies by scanning the QR code through the window, and the machine or pharmacists would dispense the drugs. People who choose “logistics delivery” could have access to real-time drug logistic information.

In the online medication consultation, people could scan the medication consultation two-dimensional code to enter a quick consultation page or select a dedicated pharmacist. Then, they presented a brief description of their medication doubts. When the consultation information was submitted, the message would be sent to the cloud consultation room on the internet. If the pharmacist was online and the number of inquiries to be accessed was below a certain threshold (e.g., 10), the pharmacist would answer the question directly. If there were many inquiries to be accessed, AI would provide references for pharmacists. If pharmacists were unavailable, the AI would reply instead.

### Qualified rate of prescription preview by AI

**FIGURE 3:**
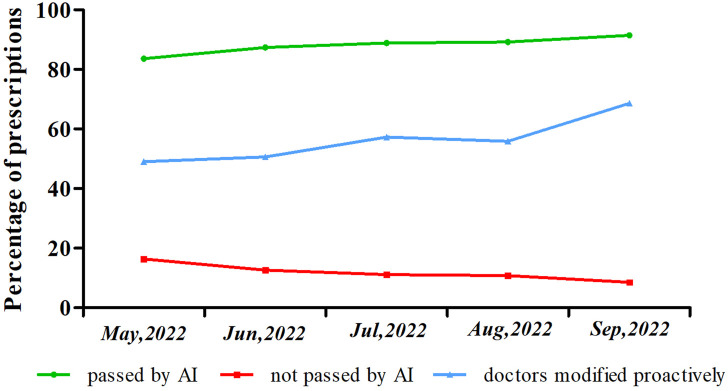
Results of prescriptions pre-reviewed by AI and modified by doctors proactively. (Green line: represents the percentage of prescriptions passed by AI; Blue line: represents the proportion of irrational prescriptions modified by doctors proactively; Red line: represents the percentage of prescriptions intercepted by AI).

Data of prescriptions pre-review from May to September 2022 was shown in Figure 3. The percentage of internet prescriptions passed by AI was always above 80%, which increased month by month. Also, leaving less than 20% of prescriptions to be handled by pharmacists. The proportion of irrational prescriptions modified proactively by doctors was also improving. Considering May 2022 as an example, the percentage of internet prescriptions passed by AI was 83.65% (Supplementary Figure S1). Among the 83.65% prescriptions passed by AI, 100% were double checked by pharmacists when dispensing. More importantly, there was zero prescription that has been rejected when dispensing in May 2022. Among the 16.35% not passed prescriptions, 49% were modified by doctors proactively and 51% were passed after the intervention of pharmacists, as shown in. For instance, the prescription of tropicamide phenylephrine eye drops was intercepted because this patient was suffering from glaucoma. To sum up, the classification of inappropriate prescriptions not passed by preview system was shown in Table 1. The most common inappropriate prescriptions intercepted by this preview system were related to the absence of a reasonable indication, long-term prescriptions (more than 4 weeks), repeated medication, incorrect routes of administration and overdose.

**TABLE 1:** The classification of inappropriate prescriptions not passed by preview system.

Classification	Proportion (%)
Absence of a reasonable indication	59.99
Long-term prescriptions	26.73
Repeated medication	4.51
Incorrect routes of administration	2.75
Overdose	2.42
Inappropriate dosing frequency	2.24
Presence of contraindication	1.36
Total	100

### Comparison on the delivery modes patients chose to pick up their medication at internet hospitals

**FIGURE 4:**
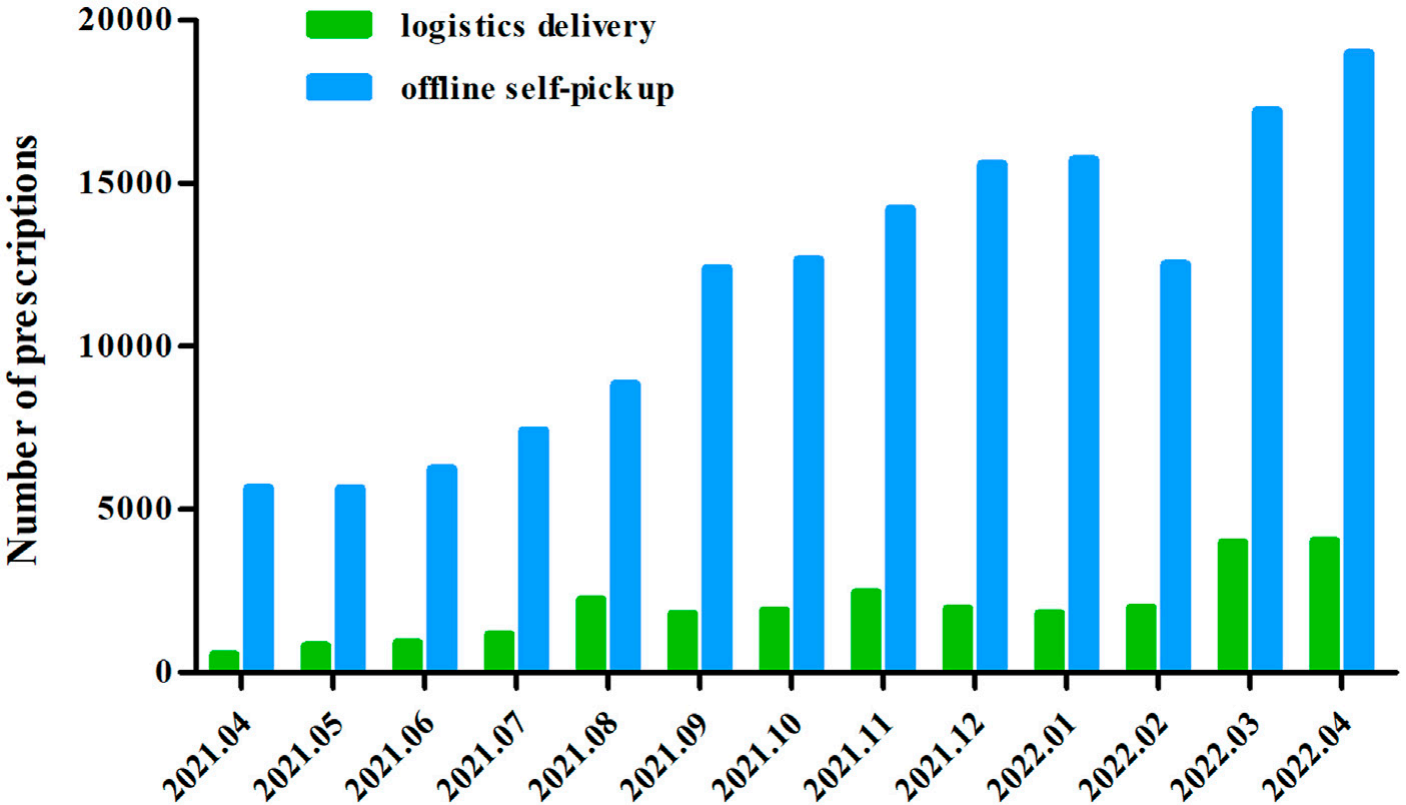
Comparison of the number of prescriptions between “logistics delivery” and “offline self-pick up.”

Prescriptions from the internet hospital had been increasing month by month since it was put into use, especially during the most severe period of the COVID-19 pandemic in Shanghai (March and April 2022). Figure 4 shows that from April 2021 to April 2022, 86% of the internet hospital prescriptions were distributed in the “offline self-pick up” mode on average. Therefore, the “offline self-pick up” was the predominant mode in the internet hospital.

### Internet hospital medication consultation

**FIGURE 5:**
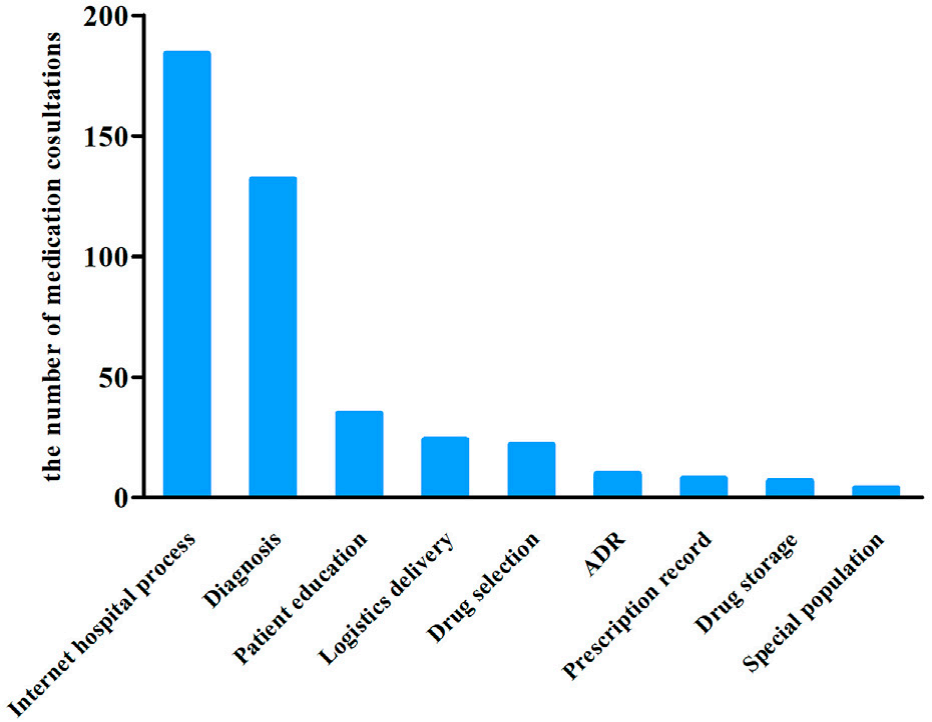
The number of medication consultation categories.

We served 426 visits of medication consultants from April 24 to 17 June 2022, during the most severe period of the COVID-19 pandemic in Shanghai. Table 2 shows the basic information about medication consultants. The mean age of patients was 29 years with the youngest age being 2 and the oldest being 89. Medication counseling for patients under 18 years old was primarily carried out by their parents. Moreover, 48% of the consultations were submitted outside working hours. Among the 426 visits, the most frequent questions were about the internet hospital dispensing process, followed by the diagnosis of disease and patient education. Figure 5 displays the detailed classification and ranking involved in the consultation content.

**TABLE 2:** Basic information of the medication consultants.

Classification	Number of consultants	No (%)
Gender		
Male	200	46.95
Female	226	53.05
Age		
<18	176	41.31
18–40	135	31.69
41–65	87	20.42
>65	28	6.57
Consultation time		
Working hours	218	51.17
Non-working hours	208	48.83

### Vision of AI-based medication consultation

**FIGURE 6:**
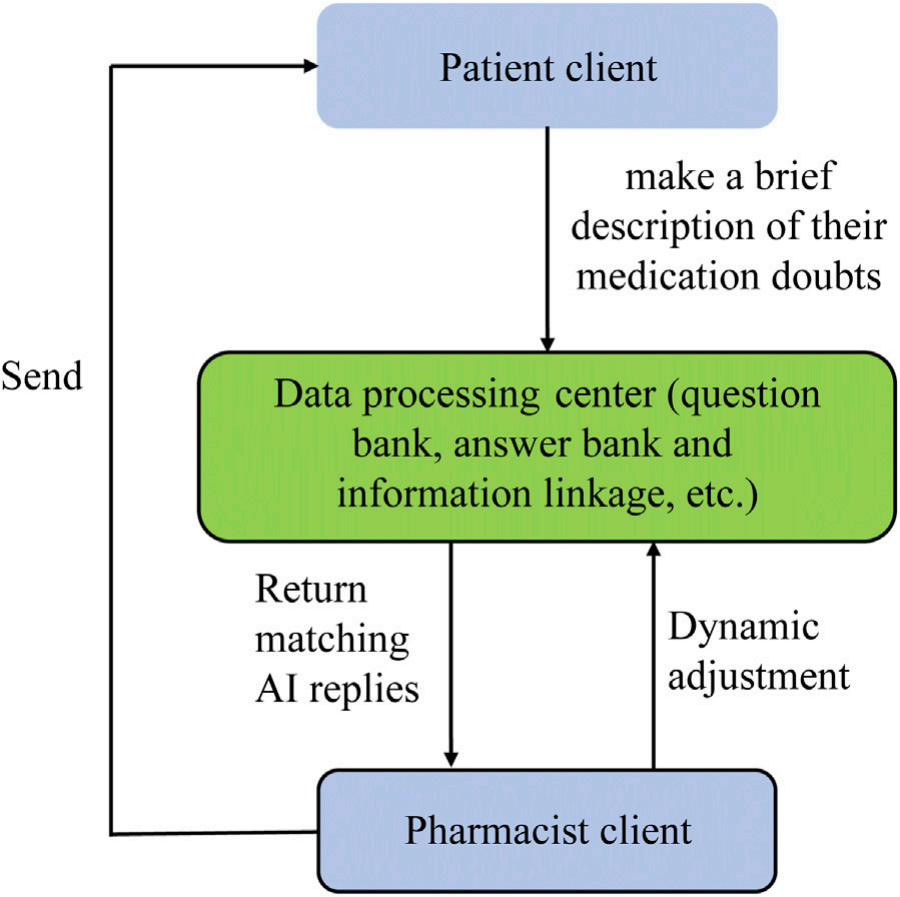
Technology roadmap of medication consultation module.

To meet the demands of medication consultation around the clock, an AI-based medication consultation mode was raised. The proposed model was an AI module that was based on the internet hospital and consisted of three parts: patient client, data processing center, and pharmacist client, as shown in Figure 6. The data processing center comprised a “question and answer bank” and logical operations that match patient questions with AI answers. The AI module library, which contained all the information in drug labels, could be dynamically adjusted and customized by users to reply more accurately and efficiently. For example, when a patient submitted an inquiry “what are the adverse reactions if the 0.01% atropine eye drops are withdrawn,” the data would be transmitted to a processing center. Then, the question would be divided into three connected entries: “0.01% Atropine drops,” “withdraw” and “Adverse reactions.” When these three entries existed side by side, AI will return “Myopia rebound may occur after withdrawal of 0.01% atropine eye drops, and it is recommended to gradually reduce the dosage when discontinuing the drug to avoid myopia rebound.” If “0.01% atropine eye drops” and “adverse reactions” just appeared together, the AI response would be “Some children may experience adverse reactions, such as photophobia and blurred vision, while taking 0.01% atropine eye drops.”

## Discussion

In this article, we reported on our initiatives in internet hospital pharmacy service combined with AI. Cloud-based medical research is growing rapidly worldwide, and its technologies are simultaneously differentiating and becoming more intelligent (Gu et al., 2020). The established AI-based internet hospital pharmacy services have four major functional segments: online drug list retrieval, prescription preview, drug dispensing and distribution, and online medication consultation, which are essential in ensuring safe and rational use of drugs as well as saving patients’ money and time.

The online drug list search function makes it more convenient for patients. Currently, most internet hospitals lack this function, which can be observed in Supplementary Table S1. Moreover, prescription preview was mainly handled manually by pharmacists in many internet hospitals. After “prescription checking specifications for medical institutions” was announced in July 2018 by the national health commission of the PRC (National health commission of the PRC, 2018), all prescriptions must be reviewed and approved before charging and dispensing. Additionally, we introduced a rational medication monitoring system and applied it to control rational drug use, which partly reduced the workload of pharmacists and increased the prescription preview efficiency. This rational medication monitoring system was widely used in prescription checks and known as a clinical decision support system (Corny et al., 2020; Hashemi et al., 2022). However, there is lack of a uniform criteria for the validation evaluation of such AI systems. In this study, prescriptions passed by AI were double checked by pharmacists and required to be 100% qualified before the launch of the system. As a result, there were no rejected prescriptions when dispensing. But for the pass rate of AI, there was no compulsory requirement. Actually, the pass rate of AI-preview was above 80%. From May to September 2022, the percentage of internet prescriptions passed by AI increased gradually, as well as the proportion of irrational prescriptions modified proactively by doctors. These results suggested that the physicians’ prescribing behavior was altered after the introduction of AI based prescription preview system.

We developed a QR code to use in offline medicine pick-up that has significantly reduced the risk of cross-infection and protect patients’ privacy. Especially, in March 2022, when Shanghai implemented the zone lockdown to control the COVID-19 epidemic, it restricted the logistics and delivery, and many patients could only entrust volunteers to pick up their medications offline from the nearest hospital (Hall et al., 2022). In traditional offline self-pickup mode, patients must present their medical insurance card, self-payment card, or medical insurance electronic certificate to pharmacists to pick up their medications. Although we have two alternatives for collecting medication, the data show that 86% of patients choose the offline pickup mode. However, for general hospitals in China, 61% of patients preferred getting their medicine through a delivery service (Ding et al., 2020). Compared with general hospitals in China, drug delivery service was also more popular during lockdown in foreign countries (Mash et al., 2021; Hammour et al., 2022). This is because that we are a specialized hospital and the characteristic medicines for disease treatment are hospital preparations. Furthermore, most hospital preparations in our hospital must be stored at 2–8°C and picked up offline.

Apart from drug dispensing, medication consultation service is another important component of internet and pharmacy service. We found that 48% of the consultations were submitted outside working hours. Presently, the traditional online medication consultation is responded to by pharmacists who are usually on duty from 8 a.m. to 5 p.m. and it is difficult for them to stay online always (Li et al., 2021). It is also difficult for pharmacists to remember all the information about indications, dosage, adverse reactions, and drug-drug interactions; it is also time-consuming to search for this information when responding. Accordingly, a combination of AI and manual mode of medication consultation will make it possible to give an immediate reply whenever necessary.

In general, the mode that combined AI with internet hospital pharmacy services could be a megatrend in developing internet hospitals and interconnecting these internet hospitals together. For example, during the COVID-19 epidemic prevention and control, to better meet the demand of residents seeking medical and pharmacy services, the “health cloud” platform (An, 2022) launched medical and pharmacy services online in April 2022 with joint efforts of the Shanghai municipal commission of health and family planning, the medical insurance platform, and a majority of hospitals in Shanghai that have launched internet hospitals. Similarly, AI enabled pharmacists to access the medical data across different health care providers (Roosan et al., 2022). Moreover, it provides people the flexibility to choose a certain doctor and pharmacist in the internet hospital to consult. However, the government guidelines are still needed for the following: 1) to supervise the whole process of internet hospital; 2) to archive the service information and realize the whole process traceability and ensure the security of relevant information; a secure and private framework must be adopted to record and administer extremely sensitive data (Mittal et al., 2022). 3) to establish a uniform charging standard for internet pharmacy service; presently, most pharmacy services are free of charge, and only a few hospitals charge fees according to the pharmacist’s title or the type of consultation.

### Limitations and future work

There are still some deficiencies of this AI system to be improved. For example, the operation interfaces of the patient client are all in Chinese, which is not friendly to foreigners. For the elder people, they often need the support from their family members to access pharmacy services in internet hospitals. What’s more, this AI system needs to be maintained regularly to avoid being unable to handle it when it goes beyond the settings. Finally, a new AI-based pharmacy service function such as “medication housekeeper” will be developed in the near future, including medication reminders (WeChat push message, short messaging service, AI voice call reminders, *etc.*), medication record (check-in and clock-in, medication adherence record), and medication tracking.

## Conclusion

We developed an AI-based pharmacy service mode of internet hospital. This mode realized drug list indexing, AI prescriptions reviewing, multiple medicine delivery methods, medication consultation, and patient education. This study suggests that the AI-based internet hospital pharmacy service ensures safe and rational drug use, saves patients’ time and economic costs, and is crucial in COVID-19 epidemic prevention and control.

## Data Availability

The original contributions presented in the study are included in the article/Supplementary Material, further inquiries can be directed to the corresponding authors.
