# Supplementary material for: Artificial intelligence-based internet hospital pharmacy services in China: Perspective based on a case study
Source: Front Pharmacol. 2022 Nov 9;13:1027808. doi: 10.3389/fphar.2022.1027808 (PMC9682042; doi:10.3389/fphar.2022.1027808)
Supplement: Supplementary file 1 [file DataSheet1.docx]

**Supplementary Table 1. Comparison of internet hospital pharmacy services among Top 10 hospitals in China's best hospital ranking**

| **Hospital** | **Established time** | **Drug Catalogue Search** | **Prescription pre-review** | **Dispensing mode** | **Pharmacy consultation and whether to charge** |
| --- | --- | --- | --- | --- | --- |
| Peking Union Medical College Hospital, Chinese Academy of Medical Sciences | May 2020 | None | Manually | logistics delivery + offline Self-pick up | Yes, free of charge |
| West China Hospital, Sichuan University | February 2020 | None | Manually | logistics delivery + offline Self-pick up | Yes, charge (30 RMB) |
| Chinese PLA General Hospital | — | — | — | — | — |
| Zhongshan Hospital, Fudan University | March 2020 | None | Manually | logistics delivery + offline Self-pick up | Yes, charge (25 RMB) |
| Ruijin Hospital, Shanghai Jiaotong University School of Medicine | April 2020 | None | Manually | logistics delivery + offline Self-pick up | Yes, free of charge |
| Tongji Hospital, Tongji Medical College, Huazhong University of Science and Technology | October 2020 | None | Manually | logistics delivery + offline Self-pick up | Yes, charge (4.5 RMB) |
| Huashan Hospital, Fudan University | March 2020 | None | Manually | logistics delivery + offline Self-pick up | Yes, free of charge |
| First Affiliated Hospital of Sun Yat-sen University | October 2019 | None | Manually | logistics delivery | Yes, charge (60-120 RMB) |
| Wuhan Union Hospital | September 2019 | None | Manually | logistics delivery | Yes, charge (4.5 RMB) |
| The First Affiliated Hospital, Zhejiang University School of Medicine | February 2016 | None | Manually | logistics delivery + offline Self-pick up | Yes, charge (10-20 RMB) |


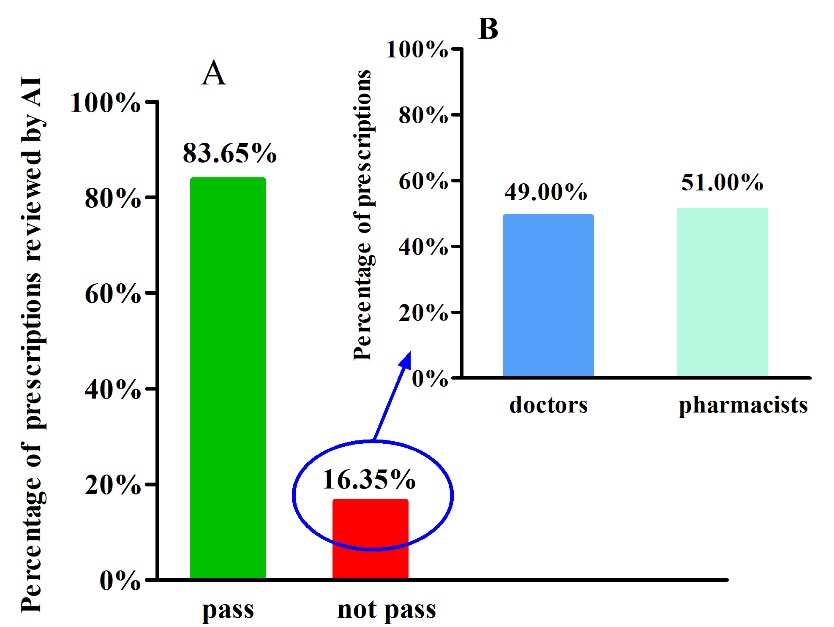


**Supplementary Figure 1.** **Results of prescriptions pre-reviewed by AI and modified by doctors proactively in May 2022.** (A: represents the percentage of prescriptions passed by AI; B: represents the proportion of irrational prescriptions modified by doctors proactively and those passed after the intervention of pharmacists).
